# Associations between Dietary Intake and Academic Achievement in College Students: A Systematic Review

**DOI:** 10.3390/healthcare5040060

**Published:** 2017-09-25

**Authors:** Tracy L. Burrows, Megan C. Whatnall, Amanda J. Patterson, Melinda J. Hutchesson

**Affiliations:** School of Health Sciences, Faculty of Health and Medicine, and Priority Research Centre in Physical Activity and Nutrition, University of Newcastle, Callaghan, NSW 2308, Australia; Tracy.Burrows@newcastle.edu.au (T.L.B.); megan.whatnall@uon.edu.au (M.C.W.); amanda.patterson@newcastle.edu.au (A.J.P.)

**Keywords:** diet, academic achievement, university students, college students, systematic review

## Abstract

The impact of diet on academic achievement is a growing area of research. The aim of this systematic review was to evaluate the current evidence examining dietary intake and academic achievement in college/university students. Eight electronic databases were searched for studies published in English to January 2016. To be included, studies must have been conducted in higher education (i.e., college, university) students, reported measures of dietary intake and academic achievement, and reported the association between these. Data were extracted using a standardised tool, and studies were assessed for methodological quality. Seven studies were included, with four rated as positive quality, and the remaining three rated as neutral. Most studies were cross-sectional (*n* = 4), and conducted in America (*n* = 5). The most common dietary outcomes were fruit and vegetable (*n* = 3), and breakfast consumption (*n* = 3). Standardised grade point average (GPA) was the most common measure of academic achievement (*n* = 4). Five studies reported small to moderate significant positive associations between diet and academic achievement, including for breakfast, regular meal consumption, and meeting national recommendations for fruit intake. This review examines the current evidence regarding diet and academic achievement in college/university students. The results demonstrate that few studies exist in this population group. Future studies should consider the use of validated dietary assessment methods, comprehensive measures of overall diet, and use standardised assessment and reporting of academic outcomes.

## 1. Introduction

Globally, the numbers of students enrolled in university or college education are high, and are increasing. In the USA, 20.2 million students were enrolled in college in 2014, reflecting a 4.9 million increase since 2000 [1]. In the UK, there were 2.3 million university students in 2016, 17% higher than in 2000 [2], while in Australia, enrolments have increased from 700,000 to over 1.2 million students in the same period [3,4]. The future success of university graduates, in terms of career, income, and associated health and quality of life, is significantly influenced by academic achievement while at university [5,6]. As such, it is in the interest of both individuals and universities to determine the factors associated with higher academic achievement. 

The effects of a range of health behaviours and indicators on academic achievement in university students have previously been established, for example, excessive alcohol use, sleep deprivation, and poor mental health status have all been shown to be detrimental to academic achievement [7,8]. The link between diet and academic achievement, however, has received much less attention in this population group. This is an important area for research, given the wealth of conferring studies demonstrating unhealthy diets of university students [9,10,11]. Understanding the potential association between dietary intake and academic achievement may help in the development of effective interventions to improve university students’ eating habits. Previous nutrition interventions targeting university students have been largely ineffective [12,13], therefore, interventions that consider unique motivators for behaviour change are warranted in this target group. Improving academic achievement may potentially motivate university and college students to change their eating behaviours.

In other population groups, such as children and adolescents, it has been demonstrated that dietary intake does influence academic achievement [14,15,16]. Mostly, existing studies have focused on breakfast consumption, with evidence showing that more frequent consumption, and higher nutritional quality of breakfast, are positively associated with academic achievement [16]. A recent systematic review in school aged students (age range 5–18 years) assessed a broader range of dietary components and behaviours with measures of academic achievement [15]. The previous review found regular breakfast consumption, higher consumption of fruit, vegetables, and certain micronutrients, including folate and iron, and lower consumption of junk foods, were all associated with higher academic achievement [15]. 

As to why diet may be linked with academic achievement, the observed associations are in line with the knowledge that various dietary components, including micronutrients such as folate, iron, and omega 3, have essential roles in brain development and functioning [17]. Furthermore, the brain requires significant and regular amounts of energy to function optimally [17]. The associations between higher consumption of nutrient rich foods, such as fruits and vegetables, and lower consumption of nutrient poor foods, such as junk foods, could then be explained by higher intakes of essential micronutrients. In addition, the associations of consuming breakfast and regular meals with higher academic achievement could be that more frequent and regular eating occasions provide a vehicle for the delivery of these nutrients, as well as adequate energy to fuel cognitive function [18]. In particular, studies in both children and adult populations have demonstrated that individuals who consume breakfast regularly have higher intakes of a range of micronutrients, including folate and iron [19,20]. Other factors known to influence academic achievement and/or diet should also be acknowledged here, such as socio-economic status (SES), gender, and other health behaviours [21,22]. For example, lower SES is associated with both lower academic achievement and poorer diet quality [11,22], reflective of the availability of social and economic resources, while several health behaviours, in particular, being physically active and attaining adequate sleep, are known to improve cognitive functioning, and subsequently, academic achievement [23,24]. Overall, existing studies on this topic tend to be lacking in their consideration of these and other potential confounding factors. 

Therefore, while the available evidence supports the association between a healthier diet and higher academic achievement in children and adolescents, no reviews have explored the association of diet and academic achievement in university and college students, highlighting a gap in the evidence base. As such, the aim of this systematic review was to evaluate the current evidence examining the relationship between dietary intake (e.g., nutrient and food group intake, eating patterns) and academic achievement in university and college students. 

## 2. Methods 

This review protocol was registered with PROSPERO (CRD42016036035) and the reporting adheres to PRISMA guidelines [25]. (http://www.crd.york.ac.uk/PROSPERO/display_record.asp?ID=CRD42016036035).

### 2.1. Criteria for Study Inclusion 

#### 2.1.1. Participants/Population

Participants were students enrolled in a university or college degree or equivalent, in any international location. 

#### 2.1.2. Exposure

Studies where dietary intake was measured and reported as the independent variable were included. For the purposes of this review, dietary intake included energy, macro/micronutrients, and food group intake, as well as diet quality, and dietary or eating patterns. Studies focused on dietary supplementation (e.g., iron, omega 3) or fortification were excluded. 

#### 2.1.3. Outcome

Studies where an academic achievement related outcome was measured and reported as the dependent variable were included. For the purposes of this review, academic achievement was defined as any outcome that showed the extent to which a student had achieved goals relating to activities performed for the purpose of a university or college degree or equivalent. This may have included such measures as grade point average, course completion, and exam/assessment achievement. Measures of attendance, behaviour at university or college, and cognitive skills were not included.

#### 2.1.4. Study Type

Observational studies (i.e., cohort, case-control or cross-sectional) were included. 

### 2.2. Search Strategy 

A three-step search strategy was undertaken to find published and unpublished studies in the English language, released up to 14 January 2016. An initial limited search of MEDLINE and Cumulative Index to Nursing and Allied Health Literature (CINAHL) was conducted, followed by an analysis of the text words contained in the title and abstract, and of the index terms used to describe the article. A second search using all identified keywords and index terms was then completed across eight electronic databases: MEDLINE and MEDLINE in progress, The Cochrane Library, EMBASE (Excerpta Medica Database), CINAHL, Eric, Scopus and PsycINFO. Key words and combinations used included: academic achievement; achievement; scholastic; university, tertiary; college; undergraduate, post graduate; higher education; food; behaviour; quality diet; pattern behaviour. A sample search strategy can be found in Supplementary Table S1. Thirdly, the reference lists of retrieved studies were searched for additional studies and checked for eligibility. 

### 2.3. Study Selection 

All studies identified during the database search were assessed for relevance to the review based on the information contained in the title, abstract, and description/MESH heading by two independent reviewers (TB, MH). For all studies that appeared to meet the inclusion criteria, or if this was unclear, the full article was retrieved. Papers selected for retrieval were assessed by two independent reviewers to determine inclusion (TB, MH). In cases of disagreement, a third independent reviewer made the final decision (AP). For studies deemed ineligible for inclusion in the review, the reasons for exclusion were listed (i.e., did not meet the inclusion criteria for types of studies, types of exposure, types of outcomes, and/or types of participants). 

### 2.4. Data Extraction 

Data were extracted from included papers using a standardised data extraction tool (Table 1 and Table 2) developed by the authors, including population characteristics, dietary intakes, academic achievement, and statistical associations reported between the two. Data extraction was completed by one reviewer (MH) and checked for consistency by a second reviewer (TB or AP). A third reviewer was consulted if any discrepancies in data extraction were identified (TB or AP). 

### 2.5. Risk of Bias (Quality) Assessment

Risk of bias was assessed by two independent reviewers (AP, TB). In the case of disagreement, a third independent reviewer made the final decision (MW). Risk of bias assessment was determined using the Academy of Nutrition and Dietetics Quality Criteria Checklist for Primary Research [26]. This tool assesses ten items relating to study validity, including four key items: the method of sample selection; methods of controlling for confounding factors; reliability of outcome measures; and statistical analysis. Each item was classified as present “Yes”, absent “No”, or “Unclear” for each included study, with each response then recoded as +1, 0, and −1, respectively. Studies were classified as positive quality when responses to all key validity questions were “yes” and had a score of eight or above, and neutral or negative if most answers were “no”, specifically for the key validation questions. No studies were excluded based on quality ratings.

### 2.6. Data Synthesis

Results are presented in narrative form using descriptive statistics to summarise study and sample characteristics, dietary intake, academic achievement, and the association between dietary intake and academic achievement.

## 3. Results

**Figure 1:**
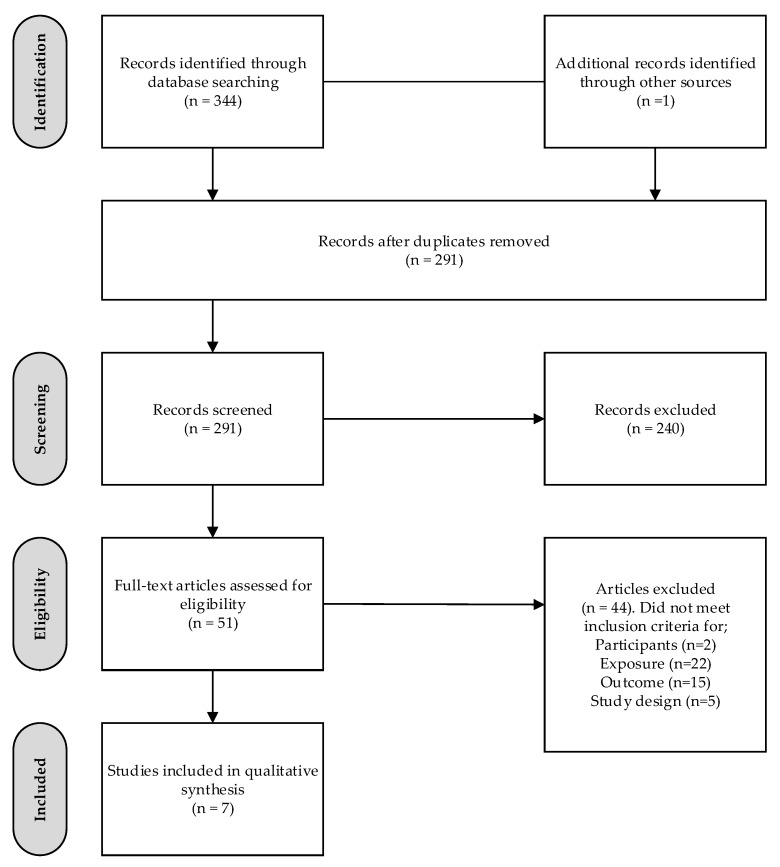
Dietary intake and academic achievement systematic review: Flow diagram.

A total of 345 studies were identified from the search strategy, with seven studies meeting the inclusion criteria (Figure 1). Study characteristics and outcomes are summarised in Table 1 and Table 2, respectively. Four cross-sectional [11,27,28,29] and three cohort studies [30,31,32] were included in the review. The majority of studies (*n* = 5) were carried out in the USA [27,28,29,30,31], one in Belgium [32], and one which reported data from 26 universities from both developed and developing nations [11]. Across the seven studies, a total of 20,107 participants were included in the review (range 101–17,789). Only four of the seven studies reported the age of participants [11,28,30,32], based on these, the mean age was 19.7 years. Two studies were carried out specifically in first year students [31,32], one in biology students [29], and one in psychology students [30]. One study was conducted exclusively in females [27], four studies were in both sexes [11,28,30,32], and the remaining two studies did not report the sex of participants [29,31]. Only three studies reported on the SES of the population, with samples having between 57% and 69% of the sample with parents who reported having a higher education [32], 54% from “wealthy/quite well off” family economic background [11], and another US study published in 1998, where the mean reported family income was US$40,000 [28]. Only two of three studies assessing SES reported accounting for this variable in the statistical analysis [11,32]. 

### 3.1. Exposure: Dietary Intake

The dietary outcome measures reported in the included studies were variable. Of the seven studies, four studies reported one dietary outcome, while the remaining three studies reported 3, 7, and 13 dietary outcomes. Fruit and vegetable intake was reported in three studies, however these were reported differently, with studies reporting number of times consumed/day [32], adequate (≥2 serves/day) or inadequate intake [11], or days/week consuming two serves [31], therefore limiting direct comparison. One study assessed eating patterns as number of regular meals/day [27], two studies assessed a range of eating habits, including frequency/week of meals and certain foods or food groups [31,32], and one study reported breakfast consumption only [29]. One study used a “nutrition” subscale [28], while another study broadly reported the “nutritional value” of diet assessed via a single questionnaire item [30], with no further details about the dietary assessment provided in these studies. 

None of the studies reported using a validated dietary assessment tool. All tools used were surveys, ranging from one to eight questions in length. Only one study reported internal reliability of the survey tool (2 short diet questions), using a Cronbach alpha statistic [11]. 

### 3.2. Outcome: Academic Achievement

Academic outcomes were most commonly reported as grade point average (GPA) (i.e., the average of grades achieved across all study subjects completed) (*n* = 4 studies) [27,28,31,32], these were obtained from college/university records in three studies and self-reported in one study. One study reported students’ self-perceived academic achievement, as well as course grade [30], while one study reported students’ self-perceived academic achievement only [11], and one study reported students’ grade on a single exam [29]. 

### 3.3. Association between Dietary Intake and Academic Achievement

The majority of studies (*n* = 5) reported statistically significant positive associations between dietary intake/behaviour and academic achievement, while two studies found no significant association. Two studies reported that those who consumed regular breakfast had higher academic achievement [29,31], with one study reporting a moderate correlation of 0.241, *p* < 0.001 [31], while the second study did not report the correlation value. A third study reported a positive correlation between GPA and regular meals, defined as 2–3 meals per day (GPA 2.9 vs. 2.6, in those not consuming regular meals) [27]. The remaining studies identified that those consuming two or more servings of fruit attained higher academic achievement, with an OR of 1.09 (95% CI 1.05, 1.13) [11], while those regularly consuming French fries or soda, or meals at university restaurants, were less likely to attend exams [32]. 

### 3.4. Risk of Bias

Risk of bias assessment is summarised in Supplementary Table S2. Four studies were rated as positive quality, and the remaining three were rated as neutral. The studies rated neutral quality tended to not report on withdrawals of participants from the study, or the statistical approach was either unclear or deemed inappropriate for the study design (e.g., comparison of outcomes with no test of statistical significance, no measurement of or adjustment for potential confounders, such as demographics).

## 4. Discussion

This is the first systematic review aiming to determine the associations between dietary intake and academic achievement in university and college students. The results are of interest to university-based health promotion practitioners, as they have the potential to guide development of nutrition interventions for university and college students. However, few studies were identified that reported dietary intake, academic achievement, and a measure of the association, with only seven studies included in the review. The majority of studies demonstrated a positive association between diet and academic achievement (*n* = 5), whereby students who reported consuming regular meals, including specifically a breakfast meal, as well as students who reported higher consumption of fruit, were found to have higher academic achievement. The included studies were rated as positive or neutral quality overall, however, key methodological limitations across the studies included the use of non-validated dietary assessment measures, and analyses failing to consider potential confounding factors known to influence academic achievement. 

The findings of this review are similar to reviews of diet and academic achievement in the school setting among children and adolescents [15,16]. Given the differences in the learning environment and learning styles in schools compared with universities, as well as the inherent differences in the life stage of students, it is notable that the findings in the two educational settings are similar. In the current review, the positive effect of breakfast consumption on academic achievement was demonstrated in five of the seven studies. Previous reviews in children and adolescents found habitual breakfast consumption, as well as nutritional quality of breakfast, were associated with greater academic achievement [15,16]. In addition, the current review also identified that consuming at least two regular meals per day was associated with higher GPAs. These findings would suggest that it is the habitual behaviour of consuming regular meals that is associated with academic achievement. Consuming regular meals, including a breakfast meal, is a healthy or recommended eating habit, and existing studies have demonstrated the relationship between increased meal frequency and greater diet quality [18]. It could therefore be postulated that the observation of higher academic achievement in students consuming regular meals might be related to greater diet quality [18,19,20]. The recent review by Burrows at el. in children and adolescents also found higher overall diet quality was associated with greater academic achievement [15]. However, there were no studies in this review that assessed overall diet quality in relation to academic achievement in university students. Given the increasing interest in the topic area, as well as in eating behaviours and health of university students more broadly, this is an important area for further investigation. 

In addition, the influence of other demographic and health characteristics, such as lower SES, and insufficient sleep, in the interaction between diet and academic achievement in university students, is currently missing from the evidence base [11,22,23,24]. This is also a criticism of the available evidence base in children and adolescents [15,16], in that studies have not considered, or adequately controlled for, factors known to influence either or both of diet and academic achievement [21,22]. The existing reviews in children and adolescent populations highlight several additional limitations which should be noted, including the use of non-validated dietary assessment methods and non-standardised subjective measures of academic achievement [15,16]. Overall, there is evidence of consistency in the findings between diet and academic achievement in university and college students, and in children and adolescents. While this strengthens the evidence for an association between the two, many of the same limitations identified in studies of children and adolescents were also identified in the current review. 

The methodological limitations of the included studies need to be considered in drawing conclusions from this review. The measures used to assess dietary intake, and to a lesser extent, academic achievement, were inadequate. None of the included studies used a validated dietary assessment tool, and they limited assessment of dietary intake to one or two food groups or eating habits, with none assessing overall diet quality. Future studies should consider more comprehensive assessment and reporting of dietary intake using validated dietary assessment tools, in order to capture overall dietary intake and more accurately determine the relationship with academic achievement. Researchers should refer to online resources such as the National Cancer Institute Dietary Assessment Primer (https://dietassessmentprimer.cancer.gov/) [33] to find appropriate, validated tools for individual studies, and refer to STROBE-Nut, an extension of the STrengthening the Reporting of OBservational studies in Epidemiology (STROBE) statement for nutritional epidemiology, to ensure appropriate reporting of dietary assessment measures (http://www.strobe-nut.org/content/strobe-nut) [34]. The majority of studies reported a standardised GPA score as the measure of academic achievement. Although some research has questioned the use of GPA to compare academic achievement, for example, because constituent grades are not weighted by level of course difficulty, overall, the evidence suggests this is a reliable measure [35,36], as well as being the most widely used. In three studies, academic achievement was self-reported by students. As with any measure of self-report, this may be associated with some bias, in particular, the studies which used a self-rating of perceived performance on a subjective scale. In terms of self-reporting GPA, however, among college students this has been demonstrated to have high correlation with recorded GPA [37], suggesting the use of self-report may be accurate in this context. 

Only three of the seven studies reported on the SES of their study sample. Of those that did, samples were primarily of higher SES, measured in terms of income or parents’ education, indicating that study samples were not diverse in this respect. Higher SES students, or those who come from higher SES backgrounds, are likely to perform better academically, and are also more likely to have healthier diets [11,22]. However, only two of the three studies to assess SES in this review also adjusted for this in statistical analyses. As such, future studies should ensure that SES is assessed, reported, and adjusted for in analyses, to improve generalisability of results. Additionally, only five of the seven studies in this review reported the gender of their study sample. Of these, four included both genders and reported to account for gender in statistical analyses. In studies of children and adolescents, academic achievement has been found to differ by gender, with females most often having higher academic achievement [14,15]. Furthermore, none of the studies in this review considered other factors which have been previously shown to influence academic achievement, i.e., potential confounders, such as ethnicity, study time, physical activity, or sleep time [23,24,38]. These are important considerations for future studies on this topic. 

This is the first review to assess the associations between diet and academic achievement in college and university students, and therefore, addresses an important gap in the evidence base. The use of a standardised review method, the ADA Quality Criteria Checklist [26], and adherence to the PRISMA guidelines also add strength to the review. The limitations of the review should also be acknowledged. Included studies were limited to those published in the English language, and did not include research theses, which may have excluded relevant studies. In addition, the majority of studies were cross-sectional in design, therefore limiting the inferences that can be made about cause and effect. 

## 5. Conclusions

This review examines the current evidence base relating dietary intake and academic achievement in university and college students. Overall, results suggest that diet may be associated with academic achievement, with the majority of studies associating more favourable dietary intake with higher academic achievement. Therefore, health promotion practitioners in the university setting should consider the positive role diet may play in students’ academic achievement when developing initiatives to promote healthy eating to students. Furthermore, health promotion practitioners may utilise the findings from the review to advocate within the university setting for the need to better support students to improve their eating habits, due to the potential impact on their academic performance. However, a limited number of lower methodological quality studies were identified, and some significant limitations of the available evidence are highlighted. Further research to more accurately determine the impact of diet on academic achievement in university and college students is warranted. Future studies should consider the use of validated dietary assessment methods, comprehensive measurement of overall dietary intake, standardised assessment and reporting of academic outcomes, and appropriate adjustment of analyses for confounding factors. 

## Figures and Tables

**Table 1:** Dietary intake and academic achievement systematic review: Study characteristics.

Study	Study Design	Population						Study Quality ^1^
		University, Country	Number	Age (Years)	% Female	Socio-Economic Status	Other Key Inclusion Criteria	
Blai et al., 1976 [27]	Cross-sectional	Harcum Junior College, USA	332	NR	100.0	NR	Students matched on CEEB Scholastic Aptitude Test (SAT) for group A & B	Neutral
Deliens et al., 2013 [32]	Cohort	Vrije Universiteit Brussel, Belgium	101	Mean: 18.0 ± 0.7	67.3	Father with higher education: 57.2% Mother with higher education: 69.0%	First year students	Positive
Larouche et al., 1998 [28]	Cross-sectional	Urban university, Boston, USA	151	Mean: 21.0 Range: 18–36	46.0	Mean family income: $40,000	None	Neutral
Peltzer et al., 2015 [11]	Cross-sectional	26 International Universities: Caribbean and South America, Sub-Saharan Africa, North Africa, East and Central Asia, South Asia, China and South-East Asia	17,789	Mean: 20.8 ± 2.8	58.7	53.8% from “wealthy/quite well off” family economic background, and 51.6% low/low–middle income countries	Universities in capital/major cities. Random sample of departments selected from universities for classes within that department to be surveyed	Positive
Phillips et al., 2005 [29]	Cross-sectional	Blinn College, TX, USA	1258	NR	NR	NR	Students enrolled in General Biology 1, in classes at 8, 9 or 10am from Spring 1993 to fall 2004	Neutral
Ruthig et al., 2011 [30]	Cohort	Public, Midwestern University, USA	203	Mean: 18.8 ± 1.5 Range: 17–24	69.0	NR	Students in introductory psychology course	Positive
Trockel et al., 2000 [31]	Cohort	Private university, USA	185	NR	NR	NR	First year students living in dormitory	Positive

NR: Not reported; ^1^ Assessed as per the Academy of Nutrition and Dietetics Quality Criteria Checklist [26].

**Table 2:** Dietary intake and academic achievement systematic review: Study results.

Study	Exposure (Measure of Dietary Intake) ^1^	Exposure (Results)	Outcome (Measure of Academic Achievement)	Outcome (Results)	Association between Dietary Intake and Academic Achievement
Blai et al., 1976 [27]	Eating patterns assessed by 8-item self-report survey (non-validated) including frequency of meals and typical foods consumed	Students categorised into: Group A: consume 2 or 3 regular meals/day. Group B: consume < 2 regular meals/day. Numbers per group not reported	Grade point average (GPA), obtained from college records	Overall GPA results not reported for full sample	Higher GPA in Group A vs. Group B (2.9 vs. 2.6), *p* < 0.01
Deliens et al., 2013 [32]	Eating habits assessed by self-report survey (non-validated). Measured 2 weeks into semester 2, with questions derived from existing surveys: Project Eat-II survey for young adults, Health and Behaviour Survey and Health Behaviour in School-aged Children survey	Mean ± SD times/week: Breakfast: 5.7 ± 2.2 Lunch: 6.6 ± 1.2 Dinner: 6.7 ± 0.9 At home with parents 3.8 ± 2.1 At student restaurant: 1.2 ± 1.5 At fast food restaurant: 0.3 ± 0.4 Other kind of restaurant: 0.3 ± 0.3 At a friend’s place 0.4 ± 0.5 French fries 0.1 ± 0.1 Fast food: 0.7 ± 0.9 Mean ± SD times/day Fruit: 1.0 ± 1.0 Vegetable: 1.2 ± 0.7 Soda: 0.8 ± 1.1	Pass/fail, based on Grade Point Average. Obtained from the University’s registration office at the end of the academic year (date not reported)	GPA 64.3 ± 9.2 Passed: 52/74 Failed: 22/74	Consumption of French fries higher in students who did not attend exams vs. students who did (0.14 ± 0.11/week vs. 0.09 ± 0.08/week *p* = 0.028). Eating at the student restaurant, soda and French fries consumption significant correlates of GPA in the univariate model only
Larouche et al., 1998 [28]	Self-report survey (non-validated): “Nutrition” subscale of Health Promoting Lifestyle Profile-II (HPLP-II) survey	Nutrition sub-scale reported only in graph	Grade Point Average, obtained from self-report survey	Overall GPA results not reported for full sample	No significant association
Peltzer et al., 2015 [11]	Fruit and vegetable intakes measured by self-report survey (non-validated), including two short diet questions (how many serves of fruit/vegetables do you eat on a typical day?)	Mean ± daily servings of fruits: 1.39 ± 1.1, vegetables: 1.66 ± 1.2, fruits and vegetables: 3.04 ± 1.9. <1 or more servings of fruit (%) 14.3%, <1 or more servings of vegetables (%) 10.3%, prevalence of <5 servings of fruit and vegetables 82.8%. Prevalence of adequate fruit (≥2 serves/day) 34.5% and vegetables (≥3 serves/day) 18.8%	Perceived academic achievement measured by self-report survey. 1-item, 5 point Likert scale (1/excellent to 5/not satisfactory)	Mean academic performance: 3.0 ± 0.9	Adequate fruit intake associated with academic achievement (OR 1.09 95%CI 1.05–1.13, *p* < 0.0001). Inadequate combined fruit and vegetable intake associated with academic achievement in unadjusted model only
Phillips et al., 2005 [29]	Self-report of breakfast consumption (non-validated; single question included on exam paper, with verbal explanation of breakfast definition provided by examiner)	Students categorised as did (65.5%)/did not (34.5%) consume breakfast on day of exam	Exam grade (A, B, C, D, E) on second major exam in General Biology	Number with each grade A: 188 B: 396 C: 236 D: 187 E: 252	Reported “significant difference” in exam achievement between those who did/did not consume breakfast
Ruthig et al., 2011 [30]	Change in self-perceived nutritional value of diet over 6 months (Time 1: end of September to Time 2: end of March), measured by self-report survey (non-validated). 1 item, 5 point Likert scale to rate nutritional value of diet (1/very poor diet, mostly junk food to 5/very good diet, no or hardly any junk food).	Mean “nutritional value”: 3.59 ± 0.80	Perception of academic achievement (1-item, 10 point Likert scale 1/very unsuccessful to 10/very successful) and course grade (provided by instructor), measured at Time 2	Overall results not reported for full sample	No significant association
Trockel et al., 2000 [31]	Eating habits assessed by 7-item self-report survey (non-validated), including frequency (days/week) consumption of supplements, breakfast, 2 serves of fruit, 3 serves of vegetables, 2 serves of “meat group”, 6 serves of “bread” group, and 2 serves of “milk” group	Not reported	Grade point average, provided by Office of Institutional Studies (at end of winter semester, date not reported)	Not reported	Significant correlation (*r* = 0.241, *p* = 0.01) between eating breakfast and GPA

^1^ Tool validation was determined if the diet assessment tool/method had a supporting reference of a diet validation study in a similar population group to that which was studied.

## Supplementary Materials

The following are available online at www.mdpi.com/2227-9032/5/4/60/s1. Table S1: Ovid Medline search strategy to identify diet and academic achievement studies in university or college students. Table S2: Risk of bias of included studies.
