# Supplementary material for: Associations between Dietary Intake and Academic Achievement in College Students: A Systematic Review
Source: Healthcare (Basel). 2017 Sep 25;5(4):60. doi: 10.3390/healthcare5040060 (PMC5746694; doi:10.3390/healthcare5040060)
Supplement: Supplementary file 1 [file healthcare-05-00060-s001.pdf]

Supplementary Table S1. Ovid Medline search strategy to identify diet and academic achievement studies in university or college students.

#	Searches	Results
1	(academic* adj5 (behavio?r* or performance* or achievement* or attain*)).mp.	5586
2	(school* adj5 (performance* or achievement* or attain*)).mp.	2802
3	(scholastic* adj5 (behavio?r* or performance* or achievement* or attain*)).mp.	344
4	Achievement/	13762
5	Educational Measurement/	30032
6	Educational Status/	42542
7	1 or 2 or 3 or 4 or 5 or 6	88706
8	Feeding Behavior/	41489
9	food intake.mp. or Eating/	65742
10	(diet* adj5 (intake or behavio?r* or quality or pattern*)).mp.	49452
11	nutrition*.mp.	273337
12	8 or 9 or 10 or 11	388610
13	((universit* or college* or tertiary or undergraduate* or postgraduate* or post graduate* or higher education) adj5 (student* or population)).mp.	39155
14	Universities/ and students/	9990
15	13 or 14	40870
16	7 and 12 and 15	57
17	limit 16 to english language	47

Explanation of search terms:

- The truncation symbol (*) retrieves all words beginning with the set of letters appearing before the symbol
- ‘adj5’ searches for both terms within five words of each other in either order.
- ‘Mp’ searches several fields at once: MP search looks in the Title, Original Title, Abstract, Subject Heading, Name of Substance, and Registry Word fields
- The forward slash symbol (/) means that the term is a valid controlled vocabulary term which has been searched in the Subject Headings field of the database.

Supplementary Table S2. Risk of bias of included studies

Study	Criteria 1	Criteria 2	Criteria 3	Criteria 4	Criteria 5	Criteria 6	Criteria 7	Criteria 8	Criteria 9	Criteria 10	Rating
Blai 1976 [1]	Y	N	Y	N	N	Y	N	Y	N	UC	Neutral
Deliens 2013 [2]	Y	Y	Y	UC	Y	Y	Y	Y	Y	Y	Positive
Larouche 1998 [3]	Y	Y	N	Y	N	Y	Y	N	Y	N	Neutral
Peltzer 2015 [4]	Y	Y	Y	N	Y	Y	Y	Y	Y	Y	Positive
Phillips 2005 [5]	Y	Y	UC	N	UC	N	UC	N	Y	UC	Neutral
Ruthig 2011 [6]	Y	Y	Y	Y	Y	Y	Y	Y	Y	N	Positive
Trockel 2000 [7]	Y	Y	Y	Y	Y	Y	Y	Y	Y	Y	Positive

[1] Assessed as per the Academy of Nutrition and Dietetics Quality Criteria Checklist [8].

The criteria assess whether 1) the research question was clearly stated; 2) selection of study participants was free from bias; 3) study groups were comparable; 4) methods for handling withdrawals were detailed; 5) blinding of study participants/personnel was used; 6) intervention/exposure were described in detail; 7) outcomes were clearly defined and measurements valid and reliable; 8) statistical analyses were appropriate for study design and outcomes; 9) conclusions were consistent with results and with consideration to biases and limitations, and 10) funding and conflicts of interest were reported and likely to have introduced bias.

References

1. Blai, B. Some biochemical correlates of academic achievement: College women--their eating habits and academic achievement. *Sci. Paedagog. Exp.* **1976**, *13*, 5-14.
2. Deliens, T.; Clarys, P.; De Bourdeaudhuij, I.; Deforche, B. Weight, socio-demographics, and health behaviour related correlates of academic performance in first year university students. *Nutr. J.* **2013**, *12*, 162.
3. Larouche, R. Determinants of college students' health-promoting lifestyles. *Clin. Excell. Nurse. Pract.* **1998**, *2*, 35-44.
4. Peltzer, K.; Pengpid, S. Correlates of healthy fruit and vegetable diet in students in low, middle and high income countries. *Int. J. Public. Health.* **2015**, *60*, 79-90.
5. Phillips, G.W. Does eating breakfast affect the performance of college students on biology exams? *J. Coll. Biol. Teach.* **2005**, *30*, 15-19.
6. Ruthig, J.C.; Marrone, S.; Hiadkyj, S.; Robinson-Epp, N. Changes in college student health: Implications for academic performance. *J. Coll. Stud. Dev.* **2011**, *52*, 307-320.
7. Trockel, M.T.; Barnes, M.D.; Egget, D.L. Health-related variables and academic performance among first-year college students: Implications for sleep and other behaviors. *J. Am. Coll. Health.* **2000**, *49*, 125-131.
8. Academy of Nutrition and Dietetics. *Evidence analysis manual: Steps in the academy evidence analysis process*; Chicago, IL, 2012.
